# On the Behavior of Different PCMs in a Hot Water Storage Tank against Thermal Demands

**DOI:** 10.3390/ma9030213

**Published:** 2016-03-21

**Authors:** Jacobo Porteiro, José Luis Míguez, Bárbara Crespo, José de Lara, José María Pousada

**Affiliations:** 1 School of Industrial Engineering, University of Vigo, Campus Lagoas-Marcosende s/n, Vigo 36310, Spain; jmiguez@uvigo.es (J.L.M.); bcrespo@uvigo.es (B.C.); 2 School of Marine Engineers, Technical University of Madrid, Madrid 28040, Spain; jose.delara@upm.es; 3 Defense University Center, Naval Academy, Marín 36900, Spain; chema@cud.uvigo.es

**Keywords:** phase change material, water tank, experimental work, thermal analysis, energetic analysis

## Abstract

Advantages, such as thermal storage improvement, are found when using PCMs (Phase Change Materials) in storage tanks. The inclusion of three different types of materials in a 60 **𝓁** test tank is studied. Two test methodologies were developed, and four tests were performed following each methodology. A thermal analysis is performed to check the thermal properties of each PCM. The distributions of the water temperatures inside the test tanks are evaluated by installing four Pt-100 sensors at different heights. A temperature recovery is observed after exposing the test tank to an energy demand. An energetic analysis that takes into account the energy due to the water temperature, the energy due to the PCM and the thermal loss to the ambient environment is also presented. The percentage of each PCM that remains in the liquid state after the energy demand is obtained.

## 1. Introduction

The electricity cost increases during peak energy demand periods, in many cases rising to twice that of off-peak periods. To address this fact, different alternatives have been investigated [1]. Phase Change Materials (PCMs), due to their thermal energy and storage capacities and based on latent energy storage, are being used as a heat storage method in heating and Domestic Hot Water (DHW) systems [2]. Energy is absorbed or generated when these materials experience a phase change. This energy is stored, and normally, it is released during peak periods. Among other facilities, their use in hot water storage tanks has commonly been studied. An extensive range of PCMs can be utilized in these applications, such as paraffin, salt hydrates, sugar alcohols and fatty acids. Different encapsulation methods, as well as diverse configurations are being studied by different researchers. N. S. Dhaidan *et al.* [3] presented a review about melting and the ensuing convection of PCMs within enclosures with different shapes, including rectangular containers, spherical capsules, cylindrical vessels and annular cavities. There is a wide range of PCM melting temperatures. In most domestic systems, the necessary hot water temperature is approximately between 50 and 60 °C, so suitable PCMs for these applications must have melting temperatures around this value.

Many studies experiment with the use of PCMs in storage tanks observing their thermal behavior, showing the advantages and thermal storage improvements of including these materials in the systems studied. I. Al-Hinti *et al.* [4] explained, after an experimental investigation, how paraffin enables the storage of a quantity of energy resulting in a higher stored hot water temperature over long periods of time. M. Ali Fazilati *et al.* [5] also incorporated paraffin in the tank of a solar water heater and observed an increase in the energy storage density and the exergy efficiency. A. Barba *et al.* [6] investigated the influence of the encapsulated PCM geometry in a domestic hot water tank testing encapsulated salt hydrates in three different geometrical configurations (slab, cylindrical or spherical polyethylene containers). M. Mazman *et al.* [7] analyzed the effect of using three different PCM mixtures (paraffin and stearic acid, paraffin and palmitic acid and stearic acid and myristic acid) in a solar domestic hot water tank. Cooling and reheating experiments were done. The best results for thermal performance enhancement of the tank were reached with the mixture of paraffin and stearic acid, observing that the temperature of 14–36 liters of water could be increased by 3–4 °C in the upper part of the tank with 3 kg of PCM.

In addition, employing PCMs in domestic hot water systems allows the use of smaller tanks in comparison with larger traditional tanks without reducing the energy stored. This fact implies an advantage when the space is a consideration. However, some researchers have indicated that there are other parameters that have a major impact in reference to this [8].

Experimental studies and simulation and numerical models checked by the experimental data have been conducted. M. Ibañez *et al.* [9] presented the modelization of a domestic hot water tank with a PCM validated by a previous experimental work [10]. J. Bony *et al.* [11] developed a numerical model of water tank storage implemented in an existing TRNSYS (Transient System Simulation Tool) tank allowing the simulation of the tank with different materials and shapes of PCM modules. The potential of the model was evaluated through comparisons between simulations and measurements. D. N. Nkwetta *et al.* [12] validated a TRNSYS model for a hot water tank with PCM with the available data. The PCM type, its amount and its location within the tank were investigated. Combining the use of sensible heat and PCM, an improvement in energy storage resulted. 

PCMs for energy storage are also studied for uses other than storage tanks. Their effects on other applications [13] are also investigated, such as in the building sector [14]. Both experimental [15,16,17] and simulation [18] studies can be found in the literature. A. Trigui *et al.* [19] compared results of using different PCM composites in passive solar walls. The thermal efficiency, as well as the quantity of energy saving when using phase change materials were presented. In another study, M. Karkri *et al.* [20] investigated the heat storage properties of plaster/paraffin building material. The same authors also investigated later the thermal properties of paraffin wax/HDPE (High-density polyethylene) [21]. M. Aadmi *et al.* [22] tested epoxy resin paraffin wax as phase change material as a new energy storage system. Numerical investigations were carried out using Comsol® Multiphysics. Experimental results were also presented. 

This paper is a continuation of an experimental study [23]. The main objective of the current research is to study in detail the behaviors of three different phase change materials in a lower capacity storage tank, 60 **𝓁**, through a series of tests simulating real consumption demands. Additionally, two different methodologies are presented and compared, which simulate one large demand and two short demands, respectively. The objective is to get a conclusion about which condition is thermally better regarding the PCMs´ response. The PCMs tested have similar melting temperatures, whereas they have distinct heat storage capacities and are encapsulated in different shapes and sizes. To carry out this study, an experimental facility was designed and implemented, which is also presented here.

## 2. PCMs Used

PCMs with transition temperatures of approximately 50–60 °C were selected because this is a suitable temperature range for domestic hot water systems and for simplicity when carrying out the experiments. In order to test different geometries and considering the commercially available materials for this temperature range, three different PCMs were selected and studied in the current research. Two of them are hydrated salts (an encapsulated and a micro-encapsulated type). A vegetable-based PCM is also studied. The encapsulated hydrated salt material, called DC58, was purchased from the firm Delta Cool (Cosella-Dörken Products, Inc., Beamsville, ON, Canada) with a transition point between 58 °C and 61 °C and a heat storage capacity of 120 kJ/kg. The vegetable-based material, PT58, with a transition point of 58 °C is from Pure Temp (Pure Temp, Plymouth, MN, USA). For this material, the heat storage capacity takes a value of 237 kJ/kg. These two materials are encapsulated with similar cylindrical shapes, but different dimensions: 30 cm length and 2 cm diameter for DC58 and 20 cm length and 2.5 cm diameter in the case of PT58. The micro-encapsulated PCM was purchased from Thermusol (Salca BV, Oortmarsum, The Netherlands) and is called HD60. The transition range for this material is between 50 °C and 60 °C, and the heat storage capacity is 160 kJ/kg. The micro-encapsulation consists of multiple cylindrical micro-capsules of 1 cm in diameter and 0.3 cm in thickness, forming like a mesh. Inside these micro-capsules, the PCM is located. 

Salt hydrates (hydrated compound·nH_2_O) are some of the most studied latent heat storage materials. They are the most important group of inorganic PCMs consisting of inorganic salts containing one or multiple water molecules. Some of the properties are that they are moderately corrosive, non-toxic and non-flammable [24]. On the other hand, the main benefit of vegetable-based PCM is that it is renewable and environmentally friendly. Some other properties of the material used in this work are that it is readily biodegradable, non-toxic, consistent, has a repeatable performance over thousands of cycles and is produced from agricultural sources [25]. Thermophysical properties data are presented in Table 1, as well as the proportion relation of the material to encapsulation.

**Figure 1:**
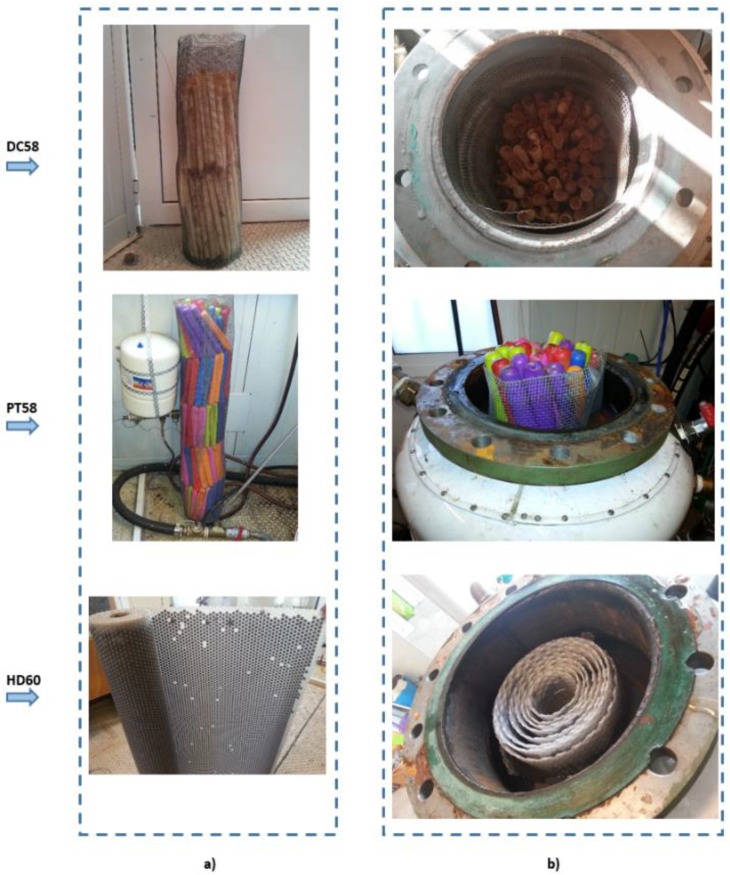
Photographs of the different PCMs studied. (**a**) Positioning; (**b**) in the test tank.

Photographs of the different PCM studied are shown below, representing the way in which they are inserted into the test tank (Figure 1a) and after they are included in it (Figure 1b).

According to the handling and storage procedures provided by the manufacturers, these materials were handled with gloves. Contact with skin and eyes was avoided, and hands were washed thoroughly after handling. The materials were kept in a tightly closed container and in a cool, well-ventilated place.

## 3. Experimental Section

### 3.1. Facility Description

**Figure 2:**
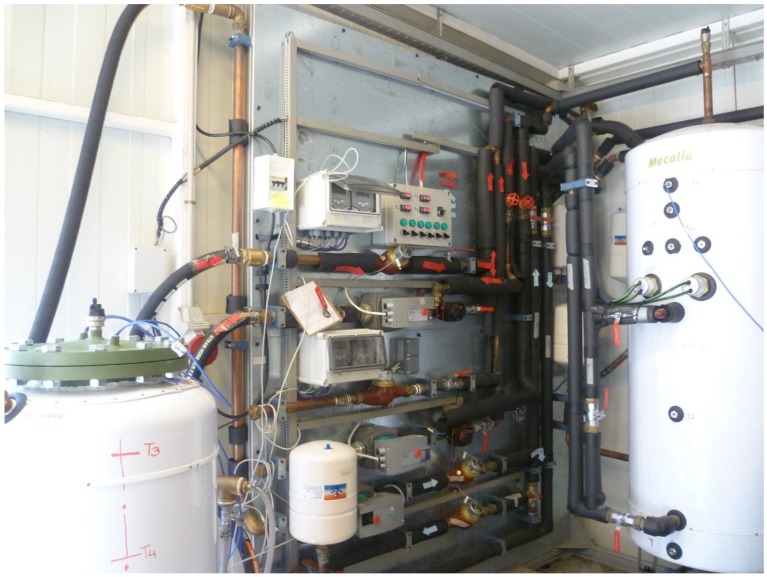
Photograph of the experimental facility.

The behaviors of PCMs in domestic hot water storage tanks are studied. For this purpose, an experimental facility (Figure 2) was built consisting mainly of two cylindrical water tanks, a 500 **𝓁** fixed inertia tank used as a hot water storage tank and a 60 **𝓁** test tank in which the different PCMs are included. Both tanks are constructed from carbon steel. 

The 60 **𝓁** test tank contains a cooling coil and a water jacket. The water jacket works as two compartments separating the fluid that circulates through it from the fluid inside the body of the tank. Consequently, three different water domains exist: the hot water that flows through the water jacket with the purpose of heating, the water that flows through the coil known as sanitary hot water (SHW) and, finally, the water inside the body of the tank where the PCMs will be placed. The tank is insulated with a layer of polyurethane of approximately 2.5 cm. Temperature pits are available in the test tank cover, allowing the control and measurement of temperature data at different heights for placing Pt-100 sensors, which have an accuracy of ± 0.2 °C and a precision of 0.03 °C. 

The energy sources used to heat the water in the 500 **𝓁** tank are a 60-kW biomass boiler and a total of six electrical resisters. A series of valves, pumps and measurement devices allows the execution under the control of the tests guaranteeing the desired water flows and temperatures. Experimental data are acquired during the tests.

Two independent circuits that allow the heating and cooling of the test tank can be differentiated in the experimental plant. The heating circuit generates temperatures in the test tank that are high enough to melt the PCM inside. This is carried out via the test tank water jacket. Hot water from the inertia tank flows through a closed circuit to the test tank water jacket. Temperatures and flows are totally controlled and regulated by the corresponded measuring devices. The cooling phase decreases the water temperature inside the test tank, solidifying the PCMs. This is done via an opened circuit through which fresh water from the network is introduced directly to the bottom part of the body of the tank, displacing the water inside the tank upwards through a superior outlet. In this way, water replacement is obtained via a piston-like movement.

**Figure 3:**
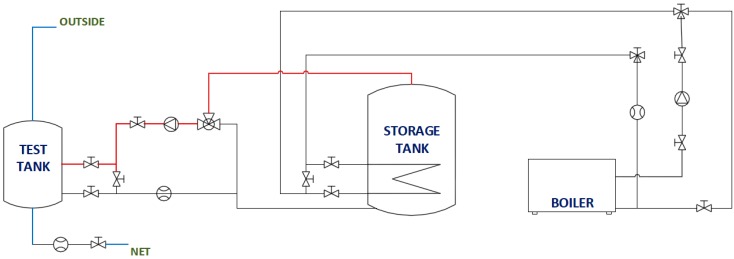
Diagram of the installation.

**Figure 4:**
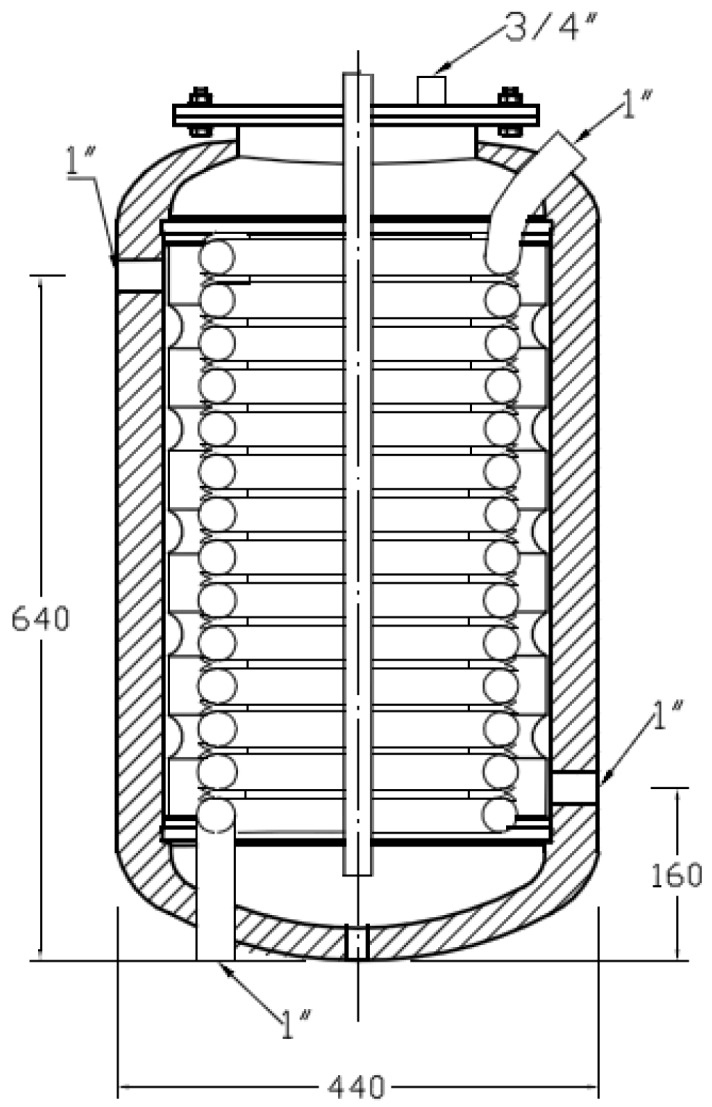
Diagram of the test tank (dimensions are in mm).

A diagram of the installation, as well as a diagram of the tank are shown in Figure 3 and Figure 4, respectively. The diagram of the installation only shows the part of the facility used in the tests presented. A more detailed description of the facility can be found in [23].

### 3.2. Method

The test methodology used in this study is intended to simulate the real operation of a domestic hot water system to analyze the response of three different phase change materials to an energy demand. Two different tests methodologies were developed that simulate two different possible real demands consisting of one large demand and two short demands. By this methodology, the behavior of the three different PCMs can be compared. In addition, the situations in which they present better thermal energy responses can be investigated to achieve the maximum use of their thermal storage capacities.

The two test methodologies consist of a total of four stages with the first two in common (Table 2). The first phase consists of heating of the test tank to achieve a temperature of approximately 80 °C in the upper layers. Heating the test tank up to this temperature, a wide margin of temperature is reached in reference to the phase change temperature of the materials, enough to take into account the stratification phenomenon. The complete melting of the PCMs inside the tank is then assured. The second phase consists of stabilizing the high temperatures reached in the heating phase. This stage has a duration of half an hour with no flow in the circuits, thus holding the test tank in a static condition. During this phase, all of the circuits are absolutely closed. There are no flows or other parameters that could affect this static condition. The third step corresponds to an energy demand simulation. In this stage, the tank is exposed to an intense cooling phase, *i.e.*, a simple demand in the first experimental methodology or two shorter demands separated by an interval of two hours corresponding to the second methodology. To obtain quick cooling, a controlled constant fresh water flow of approximately of 1200 L/h at a temperature of approximately 22 °C from the water grid is directly introduced into the body of the test tank, resulting in a replacement of the water contained in it. This value is representative of a domestic scale peak demand of about 25–30 kW. The duration of this stage was determined, after developing several previous test trials, to be a single cooling stage of 8 min or two cooling demands of 4 min each. This cooling is severe enough to completely solidify the PCMs inside the test tank. Once the third step is finished, the data acquisition continues to observe the responses of the phase change materials to these situations. This corresponds to the fourth and last phase. 

The data acquisition system registers all of the representative values of each test (flows, temperatures, pressures, *etc.*). Data are collected every second. The test tank thermal structure is represented by four different height temperatures by installing four Pt-100 sensors, from *T*1 (top temperature) to *T4* (bottom temperature) (Table 3). 

#### 3.2.1. Test Methodology Simulating One Large Demand

The third phase of this series of tests consists of a simple cooling stage simulating one large demand. As mentioned previously, after numerous tests, the duration of this energy demand was set to 8 min, which is enough to provoke the phase change of the PCMs.

Four tests (Table 4) were carried out following this methodology. A test without PCMs was previously carried out as a reference scenario. To maximize the use of the space in the inner tank, the amount of added PCM is the maximum amount that can fit, because it is not the same in all tests.

#### 3.2.2. Test Methodology Simulating Two Short Demands

A second test methodology is developed to compare the responses of the PCMs to two different possible energy demands. All of the test parameters, except those specific to the third phase, are maintained in the same conditions as the large demand methodology, so that there can be a clearer comparison between the situations.

In this way, the 8 minute-long demand is replaced by two short demands with durations of 4 min each, maintaining the total demand duration. Between the demand periods, the test tank is held static for two hours, allowing the PCMs to respond before the new energy demand. Therefore, the third phase of this methodology consists of a 4-min cooling stage, a 2-h static hold and, finally, a second 4-min cooling stage.

The quantity introduced of each PCM is the same in both methodologies. A reference test was also performed for this series of tests. The tests carried out following this second methodology are presented in Table 5.

## 4. Results and Discussion

### 4.1. Thermogravimetry and Differential Scanning Calorimetry of the PCMs

A thermal analysis was performed to check the thermal properties of each PCM. A Labsys TG-DTA/DSC (Thermogravimetry and Differential Thermal Analysis and Differential Scanning Calorimetry) from SETARAM Instrumentation (Caluire, France) with Setsoft software was used. In this way, a representative sample of about 15–20 mg was removed from the capsule for each material, and it was studied. The equipment was calibrated through the heating of various pure metals at different heating rates. Different thermal processes were applied to each material according to the manufacturers’ suggestions. Heating and cooling have been performed as slowly as possible to be near the equilibrium conditions, although the equipment limitations should be taking into account (with lower rates, much noise appears in the equipment). Enough time is given to obtain the complete melting and solidification of the material. For DC58, a heating rate of 2 °C/min from 20–70 °C was set, followed by an isotherm at 70 °C for 20 min, cooling at a rate of 2 °C/min from 70 down to 20 °C and, finally, another isotherm at 20 °C for an hour. For PT58, a heating rate of 5 °C/min from 20–80 °C was set, followed by an isotherm at 80 °C for 5 min, cooling at a rate of 5 °C/min from 80 down to 20 °C and, finally, another isotherm at 20 °C for 30 min. For HD60, a heating rate of 1 °C/min from 20–80 °C was set, followed by cooling at a rate of 1 °C/min to 50 °C for 30 min and, finally, cooling at a rate of 1 °C/min to 20 °C. 

Table 6 shows the main results obtained after the analysis, including the onset point corresponding to the melting or solidification temperature, which indicates the phase and the enthalpy.

**Figure 5:**
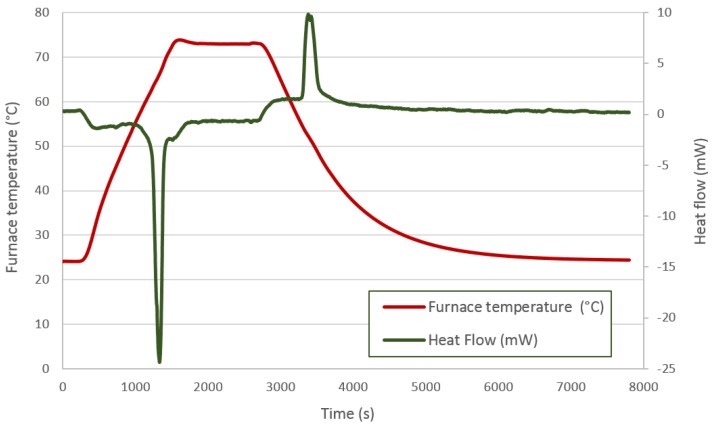
DC58 DSC analysis.

Figure 5 shows the thermal process applied to the DC58. The red line represents the furnace temperature, and the previously-mentioned ramps and isotherms can be identified in this feature. The heat flow is displayed by the green line. The first peak corresponds to an endothermic process in which the PCM absorbs a certain amount of energy related to its melting point. The second peak is representative of an exothermic process in which energy is released corresponding to the solidification of the PCM. As expected, both peak areas should be similar, as the energy absorbed by the material when melting should be released during its solidification; however, the quantity of energy released during the solidification phase is lower than that measured during the melting process.

**Figure 6:**
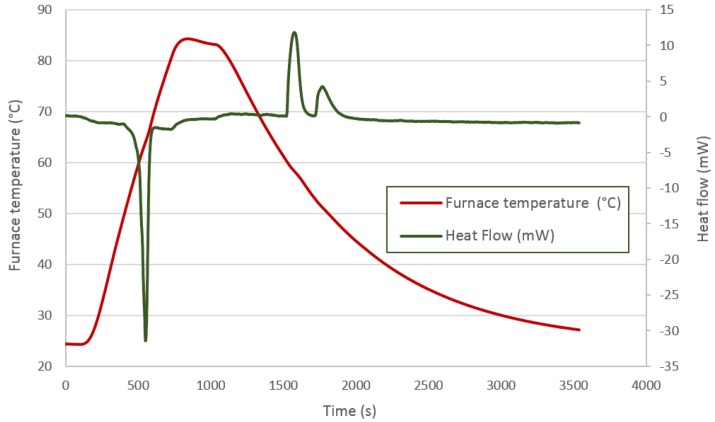
PT58 DSC analysis.

The thermal process applied to the PT58 is shown in Figure 6 and represented by the red line. As already explained, the first peak defines the energy absorbed during the melting phase. In this case, two peaks can de differentiated which could indicate a two-phase solidification. This can be attributed to the first solidification stage of the surface area followed by the second solidification stage corresponding to the inner zones. This fact can be beneficial when using the energy, as it can be available over wider time and temperature ranges. On the other hand, and more likely, this behavior can be attributed to two transitions, one freezing and one solid-solid.

**Figure 7:**
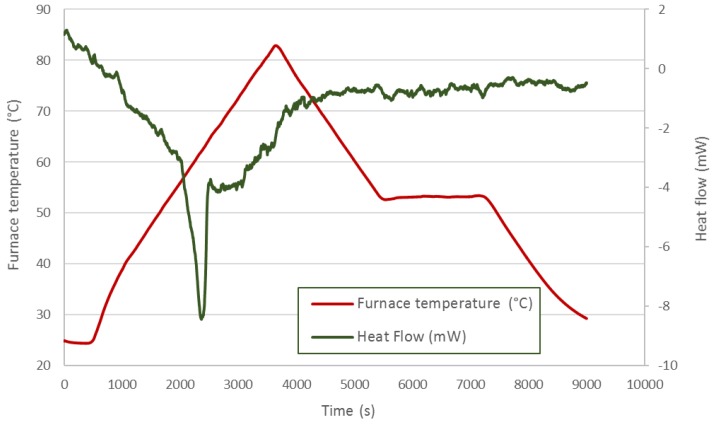
HD60 DSC analysis.

Figure 7 shows the thermal process applied to the HD60. The red line represents the furnace temperature corresponding to the thermal process. For this material, only one endothermic peak related to the melting stage is observed. A mass loss was registered for this material during the thermal process, which implies a decomposition process instead of a fusion process. This result implies that there is a chemical reaction leading to other products. This fact is corroborated because a solidification peak does not appear.

Part of the different results obtained for the melting and solidification enthalpies can be attributable to the thermal inertia of the sample. On the other hand, the fact of removing the sample from the capsule may also have led to the loss of some of the materials’ properties.

### 4.2. Study of Tank Temperature Distribution

#### 4.2.1. Case of One Large Demand

The thermal structure of the tank is studied in this section using the temperatures acquired by the four Pt-100 sensors installed at different heights in the test tank, as previously explained in the Experimental Section. Here, the four measured temperatures are plotted and compared among the different tests carried out following the methodology corresponding to one large demand. As expected, stratification in the tank can be easily observed. 

**Figure 8:**
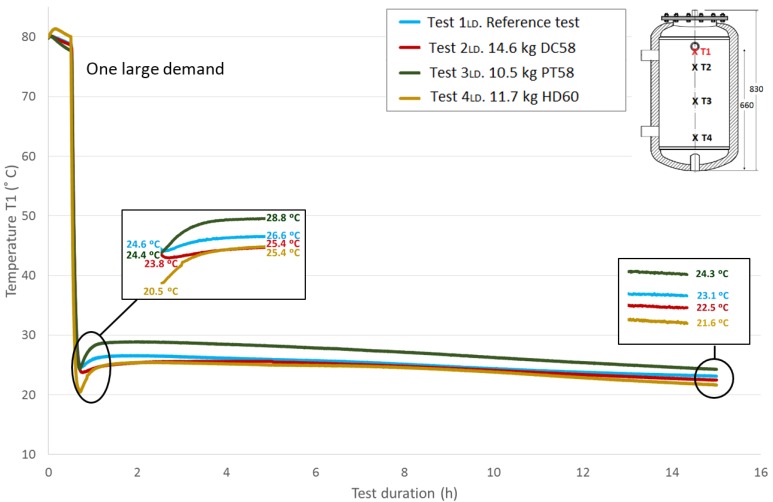
Top height water temperature (*T*1) inside the tank during one large demand experiment. (dimensions are in mm).

Figure 8 represents the progression of the *T*1 temperature values (top height) throughout the test duration for the four studied cases and the reference case; the corresponding amounts of each PCM material are included. All of the tests start at similar temperatures for easy comparison, approximately 80 °C. The first stage is not represented due to the lack of importance in the current temperature evaluation, as the main parameter to be studied is the response of the PCM after its rapid phase change (solidification), represented in this case by the large demand. The second stage (the temperatures stabilization over half an hour) corresponds to the first stage shown in the graph. During this stage, a small decrease in temperature is noted, which is not exactly the same in all of the tests due to uncontrollable test conditions, such as the environment parameters. Following the previously-explained methodology, the effect of the large demand on the temperatures is the next stage that can be seen, showing a rapid temperature drop. Both the flow and the temperature of the water introduced to the test tank in this stage were exhaustively controlled to avoid differences among tests; however, the experimental nature of this work has been taken into account. Small discrepancies related to different introduced water temperature values among the tests are corrected using the water temperature introduced in the reference test as a reference value. As shown, different temperatures were registered among tests for this sensor at the end of this stage. Immediately after, a recovery of the temperature gradients is observed, representing the response of the portion of PCM that has not reacted to the rapid demand. A zoom shot is displayed showing the lowest temperature values measured at the end of Stage III and the temperature values after the temperature recovery (peak values of Stage IV). These temperatures’ difference will be studied later. The acquisition system continues for a test duration of 15 h. A second shot shows the temperature values reached at this point, between 21.6 °C and 24.3 °C. 

**Figure 9:**
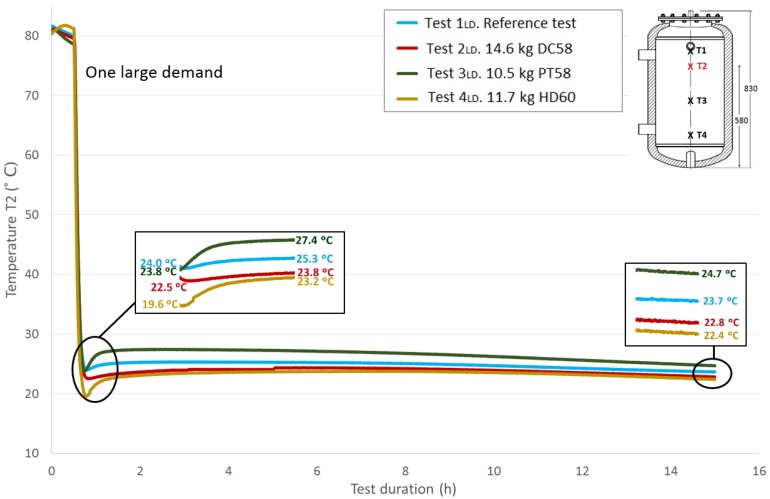
Medium top height water temperature (*T*2) inside the tank during the one large demand experiment. (dimensions are in mm).

**Figure 10:**
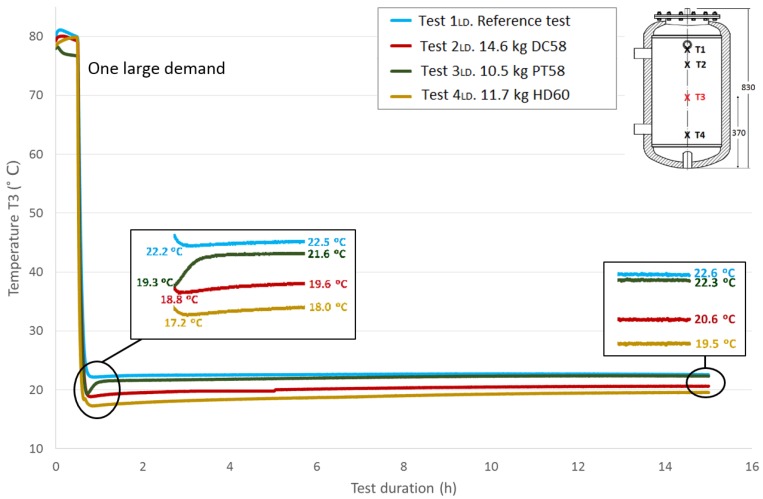
Medium bottom height water temperature (*T*3) inside the tank during the one large demand experiment. (dimensions are in mm).

**Figure 11:**
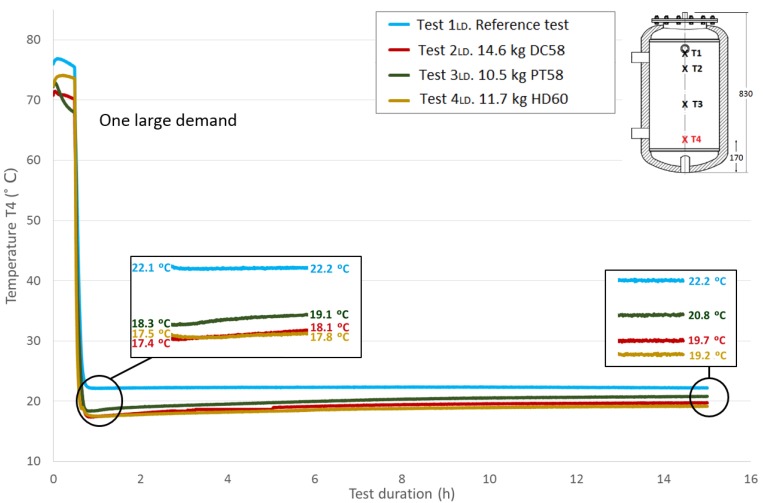
Bottom height water temperature (*T*4) inside the tank during the one large demand experiment. (dimensions are in mm).

Figure 9, Figure 10 and Figure 11 show the evolution of the temperatures at the heights *T*2, *T*3 and *T*4. The sensors height values in the test tank can be seen in a previous section or in each of the graphs. As shown in the graphs, the difference in the beginning temperatures among tests increases as the sensor height decreases because the temperatures at the beginning of the tests under similar conditions were measured by the Pt-100 sensor *T*1 as the top temperatures, which are more important in the study of storage tanks. Similar temperature progressions are found for all of the Pt-100 sensors. Smaller increases in temperatures between the end of the third stage and the peak of the fourth stage are found as the height of the Pt-100 sensors decreases.

Table 7 shows the temperature values both at the end of Stage III and at the peak of Stage IV for all of the tests and for each temperature sensor. In addition, the recovered temperature gradient is computed, which is positive in all cases. As expected, excluding the temperature gradient recovered for the temperature sensor *T*1 when DC58 is tested, regardless of the temperature sensor height, the temperature gradients are greater in the tests where the tank is filled with PCM in comparison with the reference test in which there is only water inside the tank. As previously noted, a decrease in these gradients as the sensor height decreases is also detected. The difference between the temperature gradient recovered in the tests with PCM and in the reference case can be attributed to the PCM response; therefore, the effect of including PCM in the test tank seems to have an effect on the temperature recovery. The smallest temperature gradient is recovered for DC58, which is even smaller than the one obtained in the reference case at height *T*1. A greater impact is observed when testing PT58 and HD60. Very similar temperature gradients are recovered at the *T*1 sensor, 4.4 °C and 4.9 °C for PT58 and HD60, respectively, and the same gradient of 3.6 °C for the sensor *T*2. Smaller temperature gradients are measured at the two sensors placed at the bottom of the tank.

#### 4.2.2. Case of Two Short Demands

In this section, the thermal structure of the tank corresponding to the two short demands methodology is studied. 

**Figure 12:**
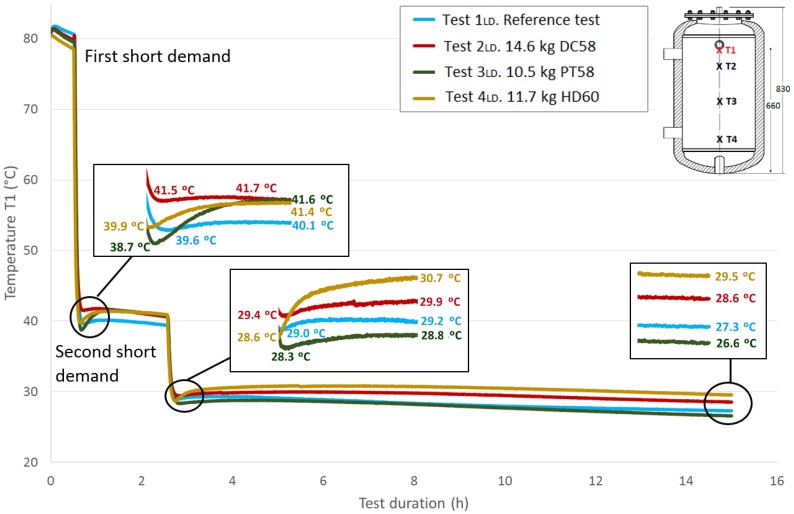
Top height water temperature (*T*1) inside the tank during the two shorts demands experiment. (dimensions are in mm).

Figure 12 shows the *T*1 temperature evolution throughout the test duration for the four cases studied. As previously explained, a value of approximately 80 °C for this sensor is taken at the start of the tests under similar conditions. The first stage shown in the graph corresponds to the temperature stabilization. As already noted, a small decrease in temperatures is observed during this phase. Then, a fast temperature decrease from approximately 80 °C to approximately 40 °C can be seen due to the effect of the first short demand. A recovery of the temperature values is observed after completing the cooling stage. A zoom shot shows the recovered temperatures. The test tank is held for two hours in static conditions, and then, it is subjected to a second short demand with the same characteristics as the first one. A second recovery of the temperature values is observed after this stage, which is represented in a zoom shot. The acquisition continues for a test duration of 15 h. A third shot represents the temperature values reached at this point, between 26.6 °C and 29.5 °C. 

**Figure 13:**
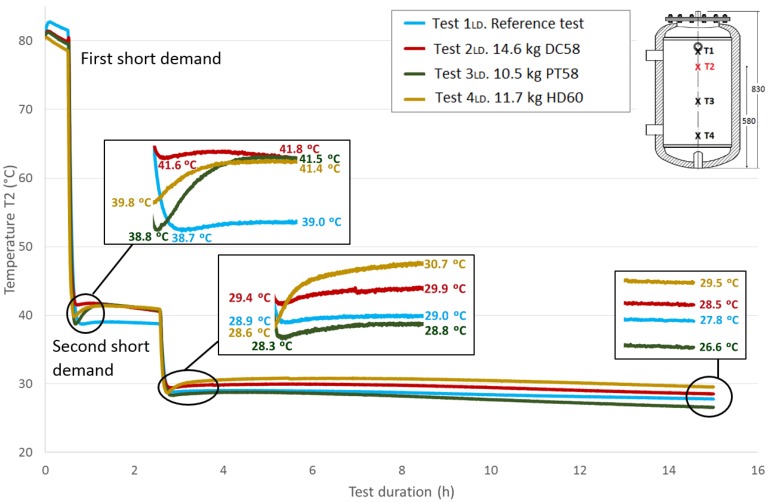
Medium top height water temperature (*T*2) inside the tank during the two shorts demands experiment. (dimensions are in mm).

**Figure 14:**
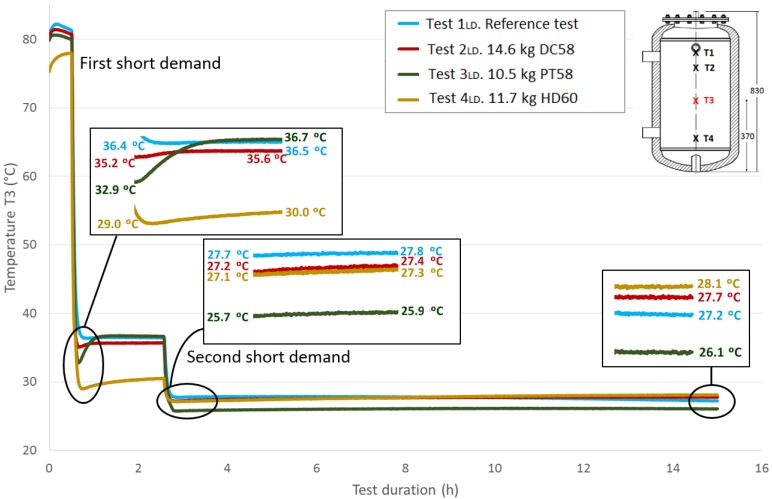
Medium bottom height water temperature (*T*3) inside the tank during the two shorts demands experiment. (dimensions are in mm).

**Figure 15:**
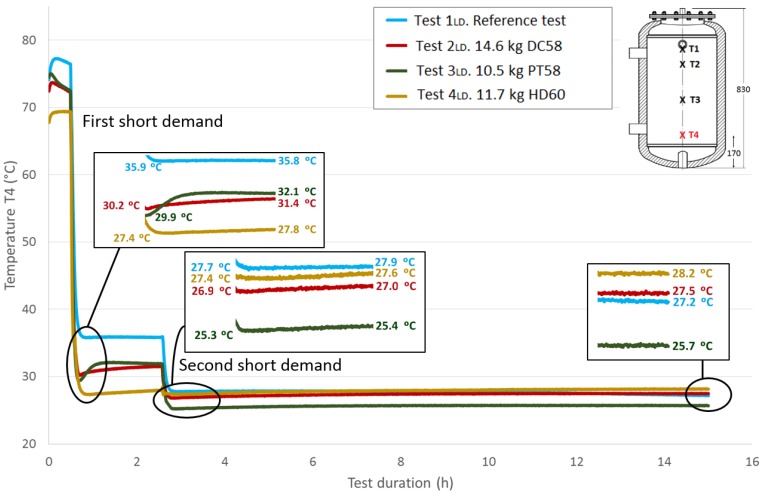
Bottom height water temperature (*T*4) inside the tank during the two shorts demands experiment. (dimensions are in mm).

Next, Figure 13, Figure 14 and Figure 15 show the progressions of the water temperatures at the heights *T*2, *T*3 and *T*4 during this experiment. 

Table 8 shows the temperature values both at the end of Stage III and at the peak of Stage IV for all of the tests, for each temperature sensor and for both short demands. The temperature gradient recovered between these tests are computed for each demand. As already observed in the previously-studied series of tests, in most cases, the temperature gradients are greater when PCM is present than in the corresponding reference test. In contrast, the decrease in the temperature gradients as the sensor height decreases, which is observed in the first series of tests, is not clearly perceived in these tests with two demands. After the first short demand, the smallest temperature gradients were recovered for DC58, as observed in the series of tests with one large demand. The largest impact is observed for PT58, which obtains temperature gradients of 2.9 °C, 2.7 °C, 3.8 °C and 2.2 °C for the sensors *T*1, *T*2, *T*3 and *T*4, respectively. On the other hand, after the second short demand, the largest temperature gradients are observed for HD60 with values of 2.1 °C, 2.1 °C, 0.2 °C and 0.2 °C for the sensors *T*1, *T*2, *T*3 and *T*4, respectively. Smaller or the same temperature gradients are obtained for the other PCMs studied, DC58 and PT58.

The total recovered temperature gradients are shown in Table 9 as the sum of the gradients obtained after both the first and the second demand. Once again, it can be clearly observed that the gradients measured in the reference case are smaller than in the corresponding tests with the PCMs. Taking into account these total values, similar gradients are registered for PT58 and HD60 for temperature sensors *T*1 and *T*2. Considerably higher values are obtained for PT58 for the temperature sensors *T*3 and *T*4. For the one large demand tests, lower temperature gradients are recovered for DC58.

Comparing the total temperature gradients obtained via both test methodologies, it can be concluded that in most cases, the temperature gradients are higher when following the one large demand methodology for sensors *T*1 and *T*2. In contrast, for the sensors *T*3 and *T*4, larger values are registered after the two short demands experiments.

### 4.3. Energetic Analysis

An energetic analysis is presented in this section to study the energy stored at different moments of the test for each case. Tests following the first methodology with one large demand are evaluated. The end of Stage II, the end of Stage III and the peak of Stage IV are the examined points, as they are considered the key times throughout the test. 

According to the four temperatures acquired during the test at different heights, the tank volume is divided into four layers. Then, the total energy is calculated as the sum of the total energy in each layer following the equation shown below (Equation (1)).

(1) $$\begin{array}{ll} E_{j}\left(t\right) & =\sum_{i=1}^{4}[m_{H_{2}O,i}*cp_{H_{2}O}*\left(T_{H2O,i}\left(t\right)-T_{amb}\right)+m_{PCM,i}\\ & *[\alpha *\left(cp_{liq\;PCM}*\left(T_{PCM,i}\left(t\right)-T_{PC}\right)+cp_{sol\;PCM}*\;\left(T_{PC}-T_{amb}\right)+\Delta h_{PCM}\right)+\left(1-\alpha \right)\\ & *cp_{sol\;PCM}*\left(T_{PCM,i}\left(t\right)-T_{amb}\right)]+\int_{t0}^{t}{\bar{UA}}_{i}*\left(T{\left(t\right)}_{H2O,i}-T_{amb}\right)*dt] \end{array}$$

As can be observed from the equation, the total energy for each layer takes into account the energy due to the water temperature, the energy due to the PCM, both in the liquid and solid state, and the thermal loss to the environment.

Some assumptions are made during the energetic analysis, which were already discussed in a previous work [23]:

- The energetic content of each section is represented by the temperature acquired by the temperature sensor placed in the center of each layer.
- The PCM material and water are evenly distributed, filling the entire tank.
- After any large heating or cooling process, the PCM is considered to exist in a state of thermal equilibrium with the surrounding water.
- When a sudden change in temperature occurs, the external layers of the PCM material are considered to follow the temperature of the water, whereas the inner core is maintained in the thermal state present at the start of the change.
- Thermal losses to the ambient environment during the test are solely related to the temperature difference between the section and the surrounding ambient environment.
- For clarity and due to its insignificant contribution, the contribution of the mass of steel in each section has been neglected.
- An ambient temperature of 17 °C is considered.

The data related to the water mass and PCM mass in each layer used during the energetic analysis are shown in Table 10. These values were computed directly proportional to each layer of the tank, which has been divided according to the second assumption. Technical data about each PCM used for this analysis were given in a previous section.

Values of U·A of 0.00033, 0.00058, 0.00084 and 0.00115 kW/°C were experimentally computed by a specific test through which the energy losses to the ambient environment were measured. This test consists of heating the test tank up to 85 °C, which was previously completely filled with water. Temperatures inside the tank were continuously recorded during days, as well as the ambient temperature. 

The results of the energetic analysis are shown in Table 11. As expected, at the end of Stage II, the total energy in the test tank achieves the highest values due to the high water temperatures in the test tank at this moment of the test and because of the energy due to the completely melted PCM. At the end of Stage II, most of the PCM has already solidified, while some remains in the liquid state. Therefore, the energy in the tank corresponds to the energy due to the water temperature and due to the solid and liquid PCM. Finally, at the point corresponding to the peak of Stage IV, the PCM has completely solidified, so there is no energy due to the PCM in the liquid state. In addition, the total energy at the end of Stage III should be the same as that at the peak of Stage IV, as the tank was isolated between both points and thermal losses to the ambient environment have been taken into account. This condition allows the calculation of the percentage of liquid PCM at the end of Stage III.

From the energy results, it can be observed that in most cases, the energy in the tank is greater in the reference case than in the tests in which PCM is present. The energy due to the PCM content is not enough to supply the energy that would be obtained if there were water instead of PCM due to the well-known high value of the specific heat of water. 

Table 12 shows the percentage of each PCM in the liquid state at the end of Stage III, which is obtained from the previous energetic analysis. These values give information about the solidification processes of each material. In this way, a very fast solidification process is detected for DC58, as only 1.2% remains in the liquid state after the large demand. In contrast, for PT58, a higher percentage, 9.7%, is still in the liquid state at the same stage of the test. In the case of HD60, this percentage is 8.8%. Initially, it could be thought that the type of encapsulation affects the solidification process; however, a clear conclusion cannot be extracted in this case, as DC58 and PT58 materials have similar encapsulation types, but they represent the fastest and the slowest solidification processes in this study. The accuracy of the results has been computed leading to a maximum uncertainty of ±1.0%.

The thermal lag that the PCMs suffer in the solidification process can be used as a heat transfer enhancement depending on the objective pursued with the use of the PCM materials.

## 5. Conclusions

In this experimental research, the inclusion of three different types of phase change materials in a 60 **𝓁** test tank is studied. The PCMs tested (DC58, PT58 and HD60) are hydrated salts and vegetable-based PCM with transition temperatures between 50 and 60 °C, but encapsulated in different ways. Two test methodologies were developed to analyze the effect of subjecting the test tank to a large energy demand or to shorter demands. For each methodology, four tests were carried out. A test with the test tank completely full of water is taken as a reference. The other three tests consist of introducing a certain amount of each PCM into the test tank. 

The results from a thermal analysis performed to check the thermal properties of each PCM are presented. The distribution of the water temperatures inside the test tank are evaluated through the continuous temperature measurement using four Pt-100 sensors installed at different heights. A temperature recovery is observed after suppressing the test tank to an energy demand representing the response of the portion of PCM that has not reacted to this demand. Smaller temperature gradients were obtained for DC58 regardless of the test methodology. A greater impact is observed when testing PT58 and HD60. In most cases, the temperature recovery is higher when following the one large demand methodology for sensors *T*1 and *T*2, while for sensors *T*3 and *T*4, larger values are registered for the two short demands methodology.

An energetic analysis is also presented using the one large demand tests. Key times throughout the tests are examined. The tank volume is divided into four layers. The energy due to the water temperature, the PCM (in both the liquid and solid states) and the thermal loss to the ambient environment are taken into account. Several assumptions are considered. From the energy results, in most cases, the energy due to the PCM content is not enough to supply the energy that would be obtained if there were water instead of PCM due to the well-known high value of the specific heat of water. 

The percentage of each PCM that remains in the liquid state after the energy demands is obtained from the energetic analysis, providing information about the solidification processes of each material. DC58 experiences a fast solidification process with only 1.2% remaining in the liquid state after the large demand. In contrast, higher percentages of 9.7% and 8.8% of the PCMs remain in the liquid state for PT58 and HD60, respectively. Initially, it could be thought that the type of encapsulation of each material affects the solidification process; however, a clear conclusion cannot be drawn, as DC58 and PT58 have similar encapsulation types, but they represent the fastest and slowest solidification processes.

## Figures and Tables

**Table 1:** PCM thermophysical properties and material-encapsulation proportion.

PCM	Transition Temperature (°C)	Latent heat (kJ/kg)	Density (kg/L)	Heat Capacity (kJ/kg·K)		Proportion (%)	
				Solid	Liquid	Material	Encapsulation
Delta Cool DC58	58–61	120	1.3	3.0	4.0	84%	16%
Pure Temp PT58	58	237	0.81	2.47	2.71	81%	19%
Thermusol HD60	50–60	160	1.3	2.5	2.5	76%	24%

Data provided from the manufacturers.

**Table 2:** Stages of the test methodologies.

Stages	One Large Demand Methodology	Two Short Demand Methodology
First phase	Heating phase up to 80 °C	Heating phase up to 80 °C
Second phase	Stabilization stage (30 min)	Stabilization stage (30 min)
Third phase	Large demand (8 min)	Short demand (4 min)
		Static period (2 h)
		Short demand (4 min)
Fourth phase	Acquisition continuation	Acquisition continuation

**Table 3:** Pt-100 sensors heights in the test tank.

Temperature	Height (mm)
*T*1	660
*T*2	580
*T*3	370
*T*4	170

**Table 4:** Tests presented simulating one large demand.

TEST	Cooling Phase	PCM	Total Mass (kg)	PCM mass (kg)
1LD (reference)	One large demand	–	–	–
2LD	One large demand	DC58	14.6	12.3
3LD	One large demand	PT58	10.5	8.5
4LD	One large demand	HD60	11.7	8.9

**Table 5:** Tests presented simulating two short demands.

TEST	Cooling Phase	PCM	Total Mass (kg)	PCM Mass (kg)
1SD (reference)	Two short demands	–	–	–
2SD	Two short demands	DC58	14.6	12.3
3SD	Two short demands	PT58	10.5	8.5
4SD	Two short demands	HD60	11.7	8.9

**Table 6:** Results from the TG-DSC analysis.

PCM	Phase Change Temperature (°C)		Enthalpy (kJ/kg)	
	Melting	Solidification	Melting	Solidification
DC58	63.1	54.4	105.3	56.2
PT58	60.2	52.7	210.3	183.0
HD60	58.6	–	91.4	–

**Table 7:** Temperature gradients recovered after the large demand at each temperature sensor.

	*T*1 (°C) (±0.2 °C)			*T*2 (°C) (±0.2 °C)			*T*3 (°C) (±0.2 °C)			*T*4 (°C) (±0.2 °C)		
	End s. * III	Peak s. IV	Temperature Gradient Recovered	End s. III	Peak s. IV	Temperature Gradient Recovered	End s. III	Peak s. IV	Temperature Gradient Recovered	End s. III	Peak s. IV	Temperature Gradient Recovered
Ref. case	24.6	26.6	2.0	24.0	25.3	1.3	22.2	22.5	0.3	22.1	22.2	0.1
DC58	23.8	25.4	1.6	22.5	23.8	1.3	18.8	19.6	0.8	17.4	18.1	0.7
PT58	24.4	28.8	4.4	23.8	27.4	3.6	19.3	21.6	2.3	18.3	19.1	0.8
HD60	20.5	25.4	4.9	19.6	23.2	3.6	17.2	18	0.8	17.5	17.8	0.3

* s.: stage.

**Table 8:** Temperature gradients recovered after the two shorts demands for each temperature sensor.

		*T*1 (°C) (±0.2 °C)			*T*2 (°C) (±0.2 °C)			*T*3 (°C) (±0.2 °C)			*T*4 (°C) (±0.2 °C)		
		End s. * III	Peak s. IV	Temperature Gradient Recovered	End s. III	Peak s. IV	Temperature Gradient Recovered	End s. III	Peak s. IV	Temperature Gradient Recovered	End s. III	Peak s. IV	Temperature Gradient Recovered
First short demand	Ref. case	39.6	40.1	0.5	38.7	39.0	0.3	36.4	36.5	0.1	35.9	35.8	-0.1
	DC58	41.5	41.7	0.2	41.6	41.8	0.2	35.2	35.6	0.4	30.2	31.4	1.2
	PT58	38.7	41.6	2.9	38.8	41.5	2.7	32.9	36.7	3.8	29.9	32.1	2.2
	HD60	39.9	41.4	1.5	39.8	41.4	1.6	29.0	30.0	1	27.4	27.8	0.4
Second short demand	Ref. case	29.0	29.2	0.2	28.9	29.0	0.1	27.7	27.8	0.1	27.7	27.9	0.2
	DC58	29.4	29.9	0.5	29.4	29.9	0.5	27.2	27.4	0.2	26.9	27.0	0.1
	PT58	28.3	28.8	0.5	28.3	28.8	0.5	25.7	25.9	0.2	25.3	25.4	0.1
	HD60	28.6	30.7	2.1	28.6	30.7	2.1	27.1	27.3	0.2	27.4	27.6	0.2

* s.: stage.

**Table 9:** Total temperature gradient recovered during the two short demands methodology.

	*T*1 (°C) (±0.2 °C)	*T*2 (°C) (±0.2 °C)	*T*3 (°C) (±0.2 °C)	*T*4 (°C) (±0.2 °C)
Ref. case	0.7	0.4	0.2	0.1
DC58	0.7	0.7	0.6	1.3
PT58	3.4	3.2	4	2.3
HD60	3.6	3.7	1.2	0.6

**Table 10:** Water and phase change material masses included in the test tank for each layer.

Layer	Water Mass, mH_2_O (kg)				Phase Change Material Mass, mPCM (kg)			
	Reference	DC58	PT58	HD60	Reference	DC58	PT58	HD60
1	6.9	5.6	5.4	5.8	–	1.4	1.0	1.0
2	12.4	10.1	9.7	10.6	–	2.5	1.8	1.8
3	17.6	14.3	13.8	14.9	–	3.6	2.5	2.6
4	23.1	18.8	18.1	19.7	–	4.7	3.3	3.4
Total	60.0	48.8	47.0	51.0	–	12.3	8.5	8.9

**Table 11:** Energetic analysis results.

		Reference Case	DC58	PT58	HD60
End of Stage II	**Total energy (lost to the ambient environment and in the tank)**	**15,099.86**	**15,407.04**	**14,546.27**	**15,453.37**
	Energy lost to the ambient environment (kJ)	286.89	250.50	244.42	297.41
	Energy in the tank (kJ)	14,812.96	15,156.54	14,301.85	15,155.96
	Due to the water (kJ)	14,812.96	11,426.97	11,073.63	12,434.18
	Due to the PCM in the solid state (kJ)	0.00	0.00	0.00	0.00
	Due to the PCM in the liquid state (kJ)	0.00	3729.57	3228.22	2721.78
End of Stage III	**Total energy (lost to the ambient environment and in the tank)**	**1783.00**	**938.70**	**1314.67**	**838.15**
	Energy lost to the ambient environment (kJ)	326.21	267.88	263.09	321.95
	Energy in the tank (kJ)	1456.79	670.82	1051.58	516.20
	Due to the water (kJ)	1456.79	529.59	674.25	253.68
	Due to the PCM in the solid state (kJ)	0.00	94.66	65.03	24.16
	Due to the PCM in the liquid state (kJ)	0.00	46.57	312.30	238.36
Peak of Stage IV	**Total energy (lost to the ambient environment and in the tank)**	**1783.00**	**938.70**	**1314.67**	**838.15**
	Energy lost to the ambient environment (kJ)	323.37	236.71	236.72	321.90
	Energy in the tank (kJ)	1459.64	701.99	1077.95	516.25
	Due to the water (kJ)	1459.64	570.84	959.12	452.93
	Due to the PCM in the solid state (kJ)	0.00	131.15	118.83	63.32
	Due to the PCM in the liquid state (kJ)	0.00	0.00	0.00	0.00
Recovered energy in the tank after the large demand (kJ)		2.85	31.17	26.37	0.05

**Table 12:** Percentages of the PCMs that remain in the liquid state at the end of Stage III (± 1.0%).

	DC58	PT58	HD60
PCM in the liquid state at the end of Stage III	1.2%	9.7%	8.8%

## Abbreviations

**Nomenclature**			
*A*	Area (m^2^)	*Subscripts*	
*cp*	Specific heat at constant pressure (kJ/kg K)	*amb*	Ambient
*E*	Energy (kJ)	*H_2_O*	Water
*∆h*	Heat storage specific capacity (kJ/kg)	*i*	Layer number
*m*	Mass (kg)	*liq*	Liquid
*t_o_*	Initial time (s)	*sol*	Solid
*t*	Time (s)	**	
*T*	Temperature (°C)	*Greek symbols*	
*U*	Overall heat transfer coefficient (kW m^2^/°C)	*α*	Fraction of PCM material in the liquid state

## Abbreviations

The following abbreviations are used in this manuscript:

LD: Large Demand
PC: Phase Change
PCM: Phase Change Material
SD: Short Demand
TG-DSC: Thermogravimetry and Differential Scanning Calorimetry
